# Detection and mapping of QTL for temperature tolerance and body size in Chinook salmon (*Oncorhynchus tshawytscha*) using genotyping by sequencing

**DOI:** 10.1111/eva.12147

**Published:** 2014-03-04

**Authors:** Meredith V Everett, James E Seeb

**Affiliations:** School of Aquatic and Fishery Sciences, University of WashingtonSeattle, WA, USA

**Keywords:** body size, Chinook salmon, linkage map, quantitative trait loci, restriction-site-associated DNA sequencing, single nucleotide polymorphism, temperature

## Abstract

Understanding how organisms interact with their environments is increasingly important for conservation efforts in many species, especially in light of highly anticipated climate changes. One method for understanding this relationship is to use genetic maps and QTL mapping to detect genomic regions linked to phenotypic traits of importance for adaptation. We used high-throughput genotyping by sequencing (GBS) to both detect and map thousands of SNPs in haploid Chinook salmon (*Oncorhynchus tshawytscha*). We next applied this map to detect QTL related to temperature tolerance and body size in families of diploid Chinook salmon. Using these techniques, we mapped 3534 SNPs in 34 linkage groups which is consistent with the haploid chromosome number for Chinook salmon. We successfully detected three QTL for temperature tolerance and one QTL for body size at the experiment-wide level, as well as additional QTL significant at the chromosome-wide level. The use of haploids coupled with GBS provides a robust pathway to rapidly develop genomic resources in nonmodel organisms; these QTL represent preliminary progress toward linking traits of conservation interest to regions in the Chinook salmon genome.

## Introduction

Impacts of global climate change on aquatic species are difficult to anticipate (Crozier et al. 2008). The warming of aquatic ecosystems may impact physiology, migration, and food webs; and organisms and populations are likely to react with both plastic and adaptive responses (Lundsgaard-Hansen et al. 2013; Piou and Prevost 2013). Thus far however, the role of adaptation in ecological responses to anthropogenic change has largely been ignored (Smith and Bernatchez 2008). Studies of genetic and physiological mechanisms of adaptation are needed to predict climate effects on ecosystems and inform conservation management (Portner and Farrell 2008; Whitehead 2012). Individual populations within species of Pacific salmon exhibit diverse responses to climate regimes (Crozier and Zabel 2006; Eliason et al. 2011; Rogers et al. 2013), indicating allelic variation for genes that mediate adaptively important physiological pathways.

Climate change and predicted oceanic temperature increases may result in substantial reductions in habitat for Pacific salmon (Schindler et al. 2008). Projected habitat losses are largest in the Gulf of Alaska and western and subarctic North Pacific Ocean. Nearly complete loss of Gulf of Alaska habitat for some species in some seasons, especially Chinook salmon (*Oncorhynchus tshawytscha*) in summer, raises important policy issues for fishery managers and governments (Abdul-Aziz et al. 2011).

The impacts of climate changes in freshwater habitats are expected to be even more pronounced than those in marine habitats: freshwater fish may have fewer options to avoid temperatures that are above optimal (or lethal) than will marine fish (Breau et al. 2011; Sutton and Soto 2012). Reproductive success and even survival of spawning adults (Farrell 2009; Evans et al. 2011; Jeffries et al. 2012) and growth and behavior of juveniles may vary greatly in substantially altered thermal regimes (Sykes et al. 2009; Sutton and Soto 2012). Thermal increases are indicated in the emergence of new diseases in freshwater that threaten natural populations (Kocan et al. 2004, 2009; Kocan and Hershberger 2006). Genetic data on thermal tolerance in salmonid species are slowly accumulating (Matala et al. 2011; Quinn et al. 2011; Anttila et al. 2013), but a more thorough and comparative approach is needed to provide insight into individual and population differences and to potentially inform conservation management (Whitehead 2012).

Chinook salmon is a cornerstone species with key economic and ecological impacts throughout the North Pacific Ocean and its freshwater drainages. Chinook salmon are anadromous and semelparous; as a result, they are a major conduit for transferring nutrients from the marine environment to support freshwater and terrestrial ecosystems. Chinook salmon are caught in commercial, sport, and subsistence fisheries in North America and Asia, and they are an important food source for marine mammals including the endangered killer whale *Orcinus orca* in the Northeast Pacific Ocean (Williams et al. 2011). Uncertain fate in the face of climate change is considerably greater for organisms such as Chinook salmon that have complex migratory life cycles where selection pressures might differ greatly between marine and freshwater life stages (Crozier et al. 2008; Schindler et al. 2008).

Genotyping by sequencing is a relatively new approach (Baird et al. 2008; Hohenlohe et al. 2010; Davey et al. 2011) that has rapidly accelerated gene discovery and the acquisition of large quantities of genomic data, facilitating genome mapping and QTL studies in nonmodel organisms (Barchi et al. 2012; Houston et al. 2012; Keller et al. 2013). Miller et al. (2012) used GBS to create a dense meiotic map in rainbow trout *O. mykiss* and detected a large haplotype block responsible for rate of embryo development; Narum et al. (2013) interrogated 10 000 random SNPs in rainbow trout and detected several that were associated with survival under thermal stress. The use of dense meiotic maps has proven to be a powerful framework for study of genomic architecture and spatial structure of divergence in natural populations (Bradbury et al. 2013; Gagnaire et al. 2013b).

Our primary objective was to use GBS to create a dense meiotic map for Chinook salmon to provide a template for later comparative mapping, QTL mapping, and population genomics studies. We used a single gynogenetic haploid family to map novel SNPs and organized them into linkage groups, consistent with the haploid chromosome number for Chinook salmon. Annotations were unremarkable; however, testing for QTL in diploid families revealed thermal tolerance signals isolated to LG 11, LG 16, and LG 34. Additionally, several growth-associated QTL were isolated to other linkage groups. We believe that this combination of GBS, efficient mapping using haploids, and QTL analyses in diploids will provide a robust pathway to rapidly develop genomic resources for Chinook salmon as well as other nonmodel organisms and provide a tool for identifying vulnerable populations to inform conservation management.

## Methods

### Animals

The project design was to conduct meiotic mapping on one haploid family and QTL mapping in one pair of diploid half-sib families. However, we designed the matings with adequate redundancy, following Institutional Animal Care and Use Committee protocol 4229-01, to guarantee that sufficient haploid individuals were available given the uncertainty of the treatment.

Matings were created from individuals in the University of Washington hatchery population over the course of 2 weeks. Six females and three males were used to make pairs of half-sib families (see Table 1 for half-sib mating strategy). A portion of the gametes from each of the females was also combined with UV-irradiated sperm from one of the Chinook salmon males or a coho salmon male to create gynogenetic haploid families for genetic mapping (cf., Thorgaard et al. 1983; Spruell et al. 1999). Fin clips from all parents were collected in ethanol for DNA analysis.

**Table 1:** Mating design for the creation of haploid and diploid families.

Male parent (diploids)	Female parent (haploids and diploids)	Male parent (haploids)
M5	F9	Coho M3
	F10	
M6	F11	Coho M7†
	F12	
M7*	F13*	
	F14*†	

QTL, quantitative trait loci.

* Parents of the diploid, half-sib families used for QTL detection, F13, and F14.

† Parents of the haploid family used for creation of the linkage map, F14H.

Haploid embryos from the six families were incubated for 2 weeks and then sampled into ethanol. Sampling was performed just prior to scheduled hatch because haploid salmonids do not survive the hatching process. Scheduled hatching time, based upon incubation temperature, was predicted using the software IncubWin (http://www.pac.dfo-mpo.gc.ca/science/aquaculture/sirp/incubwin-eng.html).

The diploid families were reared for a total of 10 weeks after hatching. At that time, they were subjected to the temperature challenge for QTL analysis.

### Temperature challenge

Individuals were starved for 48 h prior to the temperature challenge. Twenty-four hours prior to the start of the experiment, the tank water temperature was allowed to gradually rise from 12 to 18°C. At the start of the trial, the temperature was raised from 18°C to 25 ± 1°C over the course of 1 h by adding heated water to the system. This temperature was then maintained for the remainder of the trial, and the individuals were monitored for loss of equilibrium. As soon as an individual lost equilibrium, it was euthanized in MS-222 and recorded as a temperature-sensitive individual. After 50 temperature-sensitive individuals were sampled from each family, an additional 50 ‘temperature tolerant’ individuals were immediately sampled and the thermal challenge ended. The total time from initiation of the temperature challenge until the 50 temperature tolerant individuals were sampled from all three pairs of half-sib families was approximately 8 h. Sampled individuals were measured for fork-length and weight, and the whole caudal fin from each individual was preserved in ethanol for DNA analysis.

### DNA extraction, genotyping, and sequencing

All DNA extractions were carried out using DNEasy-96 kits (Qiagen, Valencia, CA, USA) following manufacturer's directions. A subsample of each axillary process from adults or whole caudal fin from diploid offspring was added to the lysis buffer directly. Whole haploid embryos were dissected from the yolk and chorion and added to the lysis buffer.

To inform the selection of a family for mapping and provide validation of the UV treatment in haploid embryos, an initial 95 haploid embryos from each family (Table 1) were screened for variation at a panel of 96 EST loci using the 5′ nuclease reaction, following the methods of Seeb et al. (2009). We wanted to select families to maximize the number of segregating ESTs to provide anchors on the map; also the absence of male alleles at EST loci was a confirmation of haploidy. All of the 5′ nuclease assays used in this study originated from Smith et al. (2005a,b2005b), Campbell and Narum (2008), Clemento et al. (2011), and Larson et al. (2013).

Haploid family F14H was selected for mapping because it had the largest number of segregating ESTs and few nonhaploid offspring. (This choice also determined the pair of half-sib families, F14 and F13, for QTL analysis.) Then, an additional 142 putative haploids from haploid family F14H were genotyped; 213 true haploids identified in these two steps were retained for further analyses. Finally, an additional 50 EST loci were genotyped in the true haploid offspring. All segregating EST loci were included in the linkage map.

Restriction-site-associated DNA (RAD) sequencing, following the methods of Baird et al. (2008), was carried out on all haploid embryos, diploid progeny, and nine adults that included the three parents of the half-sib families (F13 × M7; F14 × M7) and the six additional parents from the families reared for the thermal challenge (Table 1). All nine adults underwent paired-end sequencing to provide longer templates to improve annotation (Table S1). All other samples were processed using single-end sequencing.

Genomic DNA from each individual was cut with the restriction enzyme *SbfI*. Next, barcoded Illumina adapters were ligated to the cut site. Each individual received a unique barcode within each library, allowing individuals to be separated for data analysis. Groups of up to 24 diploids or 48 haploids (Table 1) were pooled, and the pooled DNA was sheared. For single-end libraries, DNA was sheared to around 500-bp fragments, while for paired-end libraries, DNA was sheared to around 200-bp fragments. The sheared DNA was cleaned up with a Qiagen MinElute kit, and a second Illumina adapter was added. The two Illumina adapters are necessary for fragment amplification, allowing selective amplification of only those fragments which contain a *SbfI* restriction site. Finally, the libraries were amplified with PCR, cleaned up, and sequenced. All libraries were sequenced on an Illumina HiSeq 2000 at the University of Oregon, USA.

### Sequence analysis

All RAD sequence analysis and genotyping were carried out using the freely available software program *Stacks* (Catchen et al. 2011).

First, the library that included all parents was quality-filtered and all individuals separated based on their individual barcodes using the *process_radtags* command. *Process_radtags* was set to discard reads with uncalled bases or low-quality scores. Next, SNP discovery was carried out only using the RAD sequences from the female from family F14; the sequences from the additional adults were retained for later paired-end assembly and analysis (see below). The *ustacks* command was run on the female parent using default settings, allowing the program to resolve over merged tags and discard highly repetitive sequences. Next, the *cstacks* command was run with default setting on the *ustacks* output from the female parent. The resulting catalog was used for all other alignments (see below). Finally, *sstacks* was run on the female using default settings and the catalog created in the previous step.

Restriction-site-associated DNA genotyping of the haploid offspring was also carried out using *Stacks*. Haploid libraries from F14 were run through the pipeline (*process_radtags, ustacks, sstacks*) using the same procedures as carried out on the female parent. The resulting output, along with the output and catalog from the female parent, were used with the *Stacks* genotypes command. Genotypes were set to exclude any marker that was genotyped in <160 individuals (80% of total number of haploids) and provide generic output. The resulting genotype output was opened and converted to R/qtl (Broman et al. 2003) format for linkage mapping in Microsoft Excel.

Restriction-site-associated DNA genotyping of the diploid offspring along with the two additional parents from the half-sib family was carried out using *Stacks* and the same procedures as the haploid offspring and parents (*process_radtags, ustacks, sstacks, and genotypes*). The resulting genotypes were converted into the correct formatting for gridqtl in Microsoft Excel.

### Linkage mapping

Map construction was carried out using R/qtl (Broman et al. 2003), using the parameters for doubled haploids. The genotypes from the EST assays were added to the dataset from RAD sequencing. Markers with significant segregation distortion (chi-squared test, *P* < 10^−10^) were removed from all further analysis. Recombination fractions and LOD scores were calculated using the *est.rf* command. After this step, markers with no recombination were identified, and all but a single marker were removed from initial grouping. Initial linkage group formation was carried out using the *formLinkageGroups* command with an LOD threshold of 6 and a maximum recombination fraction of 0.35. Groups were examined using *plot.rf*. Initial marker order was calculated with *orderMarkers*, using the Kosambi map function. Order was confirmed using ripple with the maximum-likelihood algorithm and a window size of 6. Any discrepancies in marker order discovered through either examination of the recombination plot or ripple were corrected using *switch.markers*. Markers with no recombination were added back to the map, and the final map was exported as a text file and figures were examined using the software program mapchart.

### Paired-end assembly and annotation

Assembly of the paired-end sequences was carried out to improve annotation of markers included in the map. Paired-end sequences from the nine parents were assembled using the paired-end module provided as part of the *Stacks* program and the freely available software program, Velvet (Zerbino and Birney 2008) (v. 1.1.06). First, *ustacks* was run on each of the remaining eight parents [the additional five females and three males from the half-sib families initially selected for rearing (see above)]. Next, the list of SNPs corresponding to the genetic map was used with the paired-end module from *Stacks* on the *ustacks* output and paired-end sequence files, providing sets of corresponding paired-end sequences for each mapped RAD tag. Velvet was run on this output using default parameters and a minimum contig length of 150 bp. Resulting contigs were compared to the SwissProt database (release 2012_10) using blastx and annotated if they matched with an e-value of 10^−4^ or less. The resulting annotations were added to the linkage map.

### Comparison with other species

Sequences from each RAD tag included in the map were compared to RAD tags from rainbow trout *O. mykiss* (Miller et al. 2012) and sockeye salmon *O. nerka* (Everett et al. 2012) using stand-alone blast (v. 2.2.25). Hits that matched along the entire length of either the trout or sockeye tag (trout and sockeye tags were shorter than the Chinook tags because of the sequencing chemistry used in those studies) and contained no more than two mismatches were retained. Positions of the tags in each species map were examined for synteny among the three species.

### Analysis of variance on phenotypes

The diploid offspring from each female (F13 × M7; F14 × M7) were reared in separate tanks, introducing a confounding tank effect in growth related traits. A one-way analysis of variance (anova) was carried out on the length and weight traits to account for this effect, and the residuals from this analysis were used in the QTL analysis for length and body weight in the paternal half-sibs.

### QTL analysis for temperature tolerance, length, and weight

QTL analysis was carried out using the online software program gridqtl (Seaton et al. 2006), which utilizes the half-sib linear regression model developed by Knott et al. (1996). This model used is *y*_*ij*_* *= *a*_*i*_+*b*_*i*_*x*_*ij*_+*e* where *y*_*ij*_ is the phenotype of individual *j* from sire or dam *i, a*_*i*_ mean effect for half-sib family *i, b*_*i*_ is regression coefficient within half-sib family *i, x*_*ij*_ is the probability of inheriting a parental allele, and *e*_*ij*_ is the residual error. The data sets were analyzed two ways using the half-sib modules in the software. First, the full paternal half-sib families were analyzed using the standard parameters for all three traits included, temperature tolerance, length, and weight. Next, the offspring from each female were analyzed separately, still using the half-sib module, classifying the dam as the shared parent to examine female-linked traits. A single QTL per linkage group was specified for both analyses.

The analysis was carried out both across all of the 34 linkage groups (experiment wide) and within each linkage group (chromosome wide). *F*-statistics were calculated at a 1-cM interval on each linkage group, and the *F*-threshold for significance was determined via a permutation test with 10 000 replicates, using the method developed by Churchill and Doerge (1994) as executed in the gridqtl software (Seaton et al. 2006). *F*-thresholds for ‘significant’ QTL were set at *P* < 0.05 at the experiment-wide level and *P* < 0.01 at the chromosome-wide level. *F*-thresholds for *P* < 0.05 at the chromosome-wide level are considered ‘suggestive’. The percentage of phenotypic variance explained (PVE) by each QTL detected was calculated as PVE = 4[1–(MSE_full_/MSE_reduced_)] where MSE_full_ and MSE_reduced_ are the mean squared error of the full model and mean squared error of the reduced model (parameters fixed), respectively (Knott et al. 1996). The 95% confidence intervals for the position of each QTL were determined using 10 000 bootstraps with resampling in the gridqtl software.

## Results

### Sequencing

Prior to sequencing, all putative haploid offspring from F14 and parents (F14, F13, M7) were successfully genotyped with the EST assays (Table S1). Genotyping showed that 25 of the putative haploids were diploids, and these were excluded from further analysis. RAD sequencing was successfully carried out on the remaining 213 haploid embryos as well as all nine adults (F14, F13, M7, and six additional adults, see Methods) and the 200 diploid half-sibs offspring of F14, F13, and M7. After quality filtering, between 729 487 and 6 685 959 reads (average 2 830 029 ± 1 427 321) were obtained for each individual (Table S2).

### SNP discovery and haploid genotyping

Single nucleotide polymorphism discovery in the female parent of the haploids, F14, produced 11 427 putative SNP loci. These loci were filtered using genotypes from the haploid individuals to remove PSVs and genotyping errors (see below).

Genotyping was successfully carried out on all haploid offspring of F14 using the catalog of all 11 427 putative SNPs discovered in the female. These genotypes went through a two-stage screening process. First, markers that were fixed for the same allele in all haploid individuals (erroneous heterozygous calls in the female parent) were removed, leaving more than 7000 putative SNPs. Next any RAD marker missing genotypes in more than 53 of the 213 individuals (25%) was removed. *Stacks* defaults to doubled haploid output when only a single parent is included in the catalog; in this format, heterozygous genotype calls are converted to missing calls, so filtering RAD markers missing more than 25% of individuals removes both true errors and PSVs. After these filters, 4905 segregating SNPs remained. These were combined with the genotype data from 91 EST assays for further analysis. After combining, the complete data set was screened to remove 1461 markers with significant segregation distortion (chi-square *P* < 10^−10^). After these filters, 3535 RAD and EST SNPs remained for linkage mapping.

### Linkage mapping

**Figure 1:**
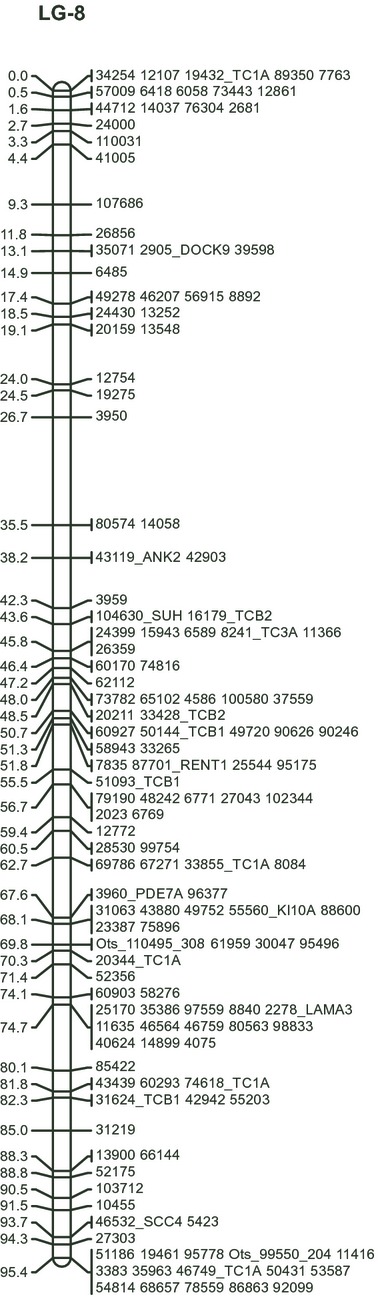
Linkage group 8 from the RAD based map for Chinook salmon. Markers beginning with ‘Ots_’ are from 5′nuclease assays. Markers which have a gene ID following the RAD number were annotated using the SwissProt database. RAD, Restriction-site-associated DNA.

Linkage mapping was successfully carried out using the doubled haploid module in R/qtl. A minimum LOD score of 6 and a maximum recombination fraction of 0.35 were used for the construction of all linkage groups. Initial linkage analysis identified a single marker with an erroneous linkage relationship. This marker was removed from further analysis, and the linkage relationships were recalculated. The final map contained 3534 SNPs, arranged in 34 linkage groups and a single unlinked marker. Each linkage group contained between 24 and 193 SNPs. Linkage groups ranged in size from 27.75 to 160.23 cM, with an average marker spacing of between 0.39 and 1.78 cM (Fig. 1, Table 2, Table S3). The total female map length was 2483.41 cM. Each linkage group also contained blocks of between two and 15 markers with no detectable recombination (Table S3, Fig. 1).

**Table 2:** Linkage summary. The number of markers, length, and average marker spacing of each of the 34 Chinook salmon linkage groups.

Linkage group	Number of markers	Length (cM)	Average marker spacing (cM)
1	182	100.06	0.55
2	167	160.23	0.96
3	193	96.39	0.50
4	179	86.11	0.48
5	165	97.51	0.59
6	151	86.07	0.57
7	153	105.34	0.69
8	144	95.35	0.66
9	135	98.75	0.73
10	142	87.41	0.62
11	125	97.96	0.78
12	126	49.53	0.39
13	124	87.09	0.70
14	101	112.65	1.12
15	120	88.79	0.74
16	88	70.23	0.80
17	91	63.79	0.70
18	86	49.07	0.57
19	88	56.25	0.64
20	90	51.95	0.58
21	84	52.63	0.63
22	107	62.72	0.59
23	85	100.35	1.18
24	85	54.66	0.64
25	60	47.03	0.78
26	83	46.08	0.56
27	58	47.57	0.82
28	61	53.16	0.87
29	72	52.71	0.73
30	47	56.27	1.20
31	45	52.74	1.17
32	46	46.52	1.01
33	24	42.66	1.78
34	26	27.78	1.07

Using *Stacks* and Velvet, 3462 contigs were successfully assembled from the paired-end sequences. These contigs were compared to the SwissProt database using blastx, and hits with an *e*-value <10^−4^ were retained. In this manner, 426 SNPs were successfully annotated (Table S4).

The 426 annotated RAD tags include a variety of biological processes and when possible were assigned GOSlim terms. Nearly half (209/426) of all annotations were transposable elements. The remaining annotations included genes involved in transcription (18), transport of proteins and ions (19), cell differentiation (11), development (6), and others (16) (Table S3). The remaining nontransposable element sequences were not assigned a process using GOSlim terms. These proteins may have unknown functions or their functions are not included in those included in the GOSlim definitions.

Restriction-site-associated DNA tags included in the map were successfully compared to previously mapped RAD tags from sockeye salmon (Everett et al. 2012) and rainbow trout (Miller et al. 2012). Forty-one tags were shared between sockeye salmon and Chinook salmon, and 81 tags were shared between rainbow trout and Chinook salmon (Table 3). These matches were spread throughout the genomes of all three species.

**Table 3:** Comparison to other species. Linkage group matches to RAD-tag-based maps in rainbow trout *O. mykiss* and sockeye salmon *O. nerka* (Everett et al. 2012; Miller et al. 2012; Palti et al. 2012).

Chinook Linkage Group	Rainbow Trout Linkage Group	Number of hits	Sockeye Salmon Linkage Group	Number of hits
1	WS16	1	One_25	1
2				
3	WS01,WS27	4	One_28	1
4	WS16, WS19	4	One_4, One_11	2
5	WS03, WS05	3	One_21, One_24	3
6	WS25	3		
7	WS06	2	One_3	1
8	WS04	3	One_9	2
9	WS07	1	One_13, One_27M	2
10	WS24	10		
11	WS15, WS22	5	One_18	1
12	WS18	2	One_9, One_13	2
13	WS13	5	One_27, One_28 M	4
14	WS23	4		
15	WS17, WS25	2	One_14	1
16	WS26	3	One_8	1
17	WS03	3	One_4	1
18	WS01	4	One_13	2
19	WS11	2	One_19	2
20			One_10	1
21	WS09	3	One_20	1
22	WS02	1	One_1	1
23	WS04, WS16	2	One_22	1
24	WS15	2	One_9, One_16	2
25	WS10	1	One_7	1
26	WS08	3	One_20	1
27	WS08	2	One_23	
28			One_6	1
29	WS10	1	One_6	1
30	WS29	1	One_21	1
31	WS06	1	One_10, One_13, One_14	3
32	WS12, WS15	3		
33				
34			One_21	1
Total		81		41

RAD, Restriction-site-associated DNA.

The final linkage map was used for QTL analysis with the diploid half-sibs. Diploid individuals were successfully genotyped for the 3472 SNPs included in the linkage map using *Stacks*. QTL detection was carried out at both the experiment-wide and chromosome-wide level. At the experiment-wide level, a *P*-value of 0.05 was significant, and at the chromosome-wide level, a *P*-value of 0.01 was significant for QTL and 0.05 was suggestive for QTL, similar to the procedure applied in Gutierrez et al. (2012).

### Thermotolerance QTL

**Figure 2:**
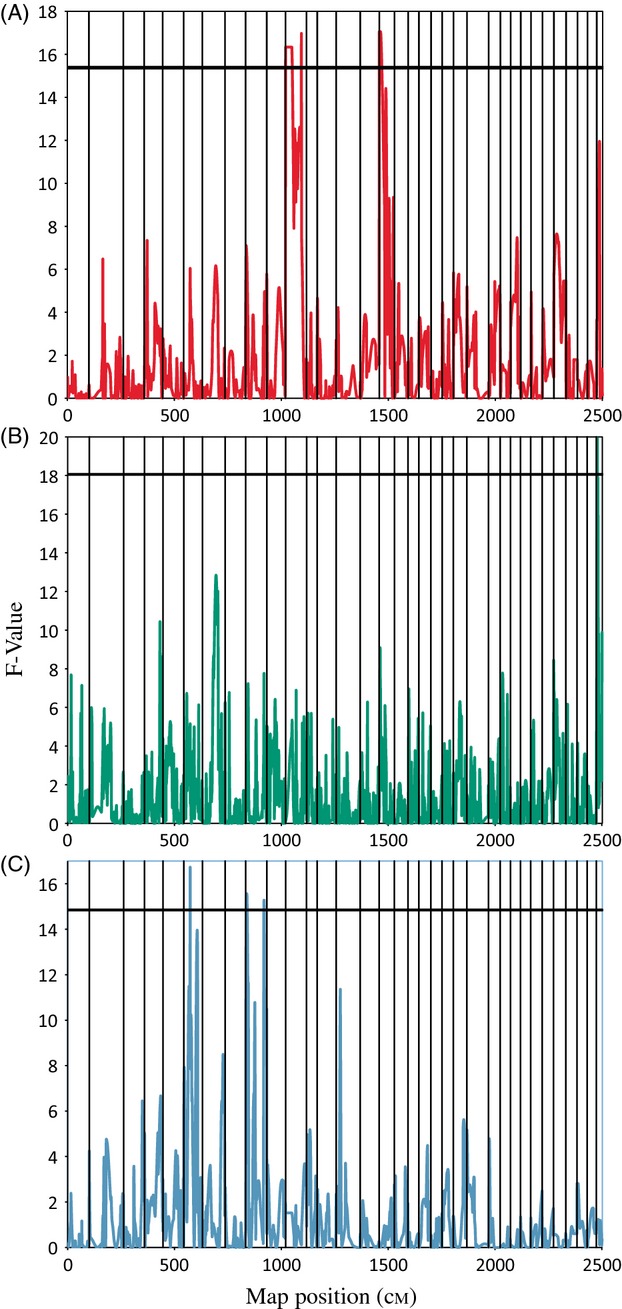
The distribution of *F*-values containing QTL significant at the experiment-wide level in the male (M7) and female (F14) parents. (A) *F*-values for thermotolerance in the male. (B) *F*-values for thermotolerance in the female F14. (C) *F*-values for body weight in the male. The dark horizontal line in all three figures is the experiment-wide (*P* < 0.05) significance threshold determined by a 10 000 permutation test (Churchill and Doerge 1994). Vertical lines designate individual linkage groups. Linkage group 11 on plot A contains two peaks, our model specified one QTL per linkage group, so only the higher of the two peaks was determined to be a QTL. QTL, Quantitative trait loci.

Within the paternal half-sib families, two male-linked QTL were found at the experiment-wide level (Fig. 2A): one QTL was significant at the chromosome-wide level, and an additional two were suggestive at the chromosome-wide level (Table 4). The male-linked QTL significant at the experiment-wide level were located on linkage groups 11 at 74 cM and 16 at 6 cM (Fig. 2A). The chromosome-wide QTL was located on linkage group 34 at 13 cM. The two suggestive QTL were located on linkage group 26 at 30 cM and linkage group 30 at 13 cM (Table 4). These QTL account for between 12 percent (suggestive QTL) and almost 30 percent (experiment-wide QTL) of the PVE (Table 4). Examining the female-linked QTL, there is a single QTL significant at the genome-wide level in female F14, located on linkage group 34 at 5 cM. There are an additional two suggestive QTL on linkage groups four and seven, located at 73 and 63 cM, respectively. These QTL accounted for between 34 and 64% of the PVE (Table 4). In female F13, no thermotolerance QTL were discovered at the genome-wide level. Additional data on each QTL for thermotolerance and body size (see below), including QTL effect, can be found in Table S5.

**Table 4:** Significant QTL. *F*-values and thresholds for significant QTL at both the chromosome-and genome-wide levels. Trait 1 is thermotolerance, two is length, and three is weight.

Linkage Group	Trait	Parent	Position (cM)	*F*	Chromosome-wide *F*-value threshold (*P* < 0.05)	Chromosome-wide *F*-value threshold (*P* < 0.01)	Experiment-wide *F*-value threshold (*P* < 0.05)	Significance	% PVE	95% C.I. (cM)
4	1	F14	73	10.44	9.71	13.89	18.05		34.82	2.0–93.0
5	2	M7	69	10.34	8.51	11.48	13.96		17.94	22.5–95.0
6	2	M7	64	11.53	7.83	10.78	13.96	^*^	20.11	23.0–67.0
6	3	M7	29	16.73	8.37	11.96	14.85	^*^^*^	29.31	4.0–64.0
6	3	F14	14	9.11	8.93	12.47	16.29		30.29	1.0–78.0
7	2	M7	95	10.79	8.34	12.33	13.96		18.75	8.0–103.0
7	1	F14	63	12.84	10.13	13.99	18.05		42.73	17.0–89.0
9	2	M7	86	10.19	8.51	11.56	13.96		17.66	1.0–88.0
9	2	F14	5	8.43	8.24	11.06	14.04		27.94	4.0–92.0
9	3	M7	5	15.56	8.86	11.85	14.85	^*^^*^	27.28	2.0–88.0
9	3	F14	5	10.04	9.09	12.49	16.29		33.46	4.0–92.0
11	1	M7	74	16.98	6.97	10.59	15.37	^*^^*^	29.73	2.0–74.0
14	2	M7	20	8.32	7.62	10.48	13.96		14.20	2.0–83.0
14	2	F14	44	8.94	8.57	11.54	14.04		29.69	1.0–112.0
14	3	M7	20	11.36	7.89	11.25	14.85	^*^	19.79	8.0–53.0
14	3	F13	74	9.86	9.35	12.76	15.82		32.87	8.0–79.0
16	1	M7	6	17.05	8.32	11.89	15.37	^*^^*^	29.86	1.0–67.0
18	3	F13	7	9.53	8.39	12.00	15.82		31.72	0.0–35.0
23	2	F14	60	9.08	8.18	11.18	14.04		30.19	0.0–62.0
26	1	M7	30	7.48	7.30	10.60	15.37		12.61	0.0–44.0
30	1	M7	13	7.65	6.18	9.83	15.37		12.93	0.0–53.0
34	1	M7	13	11.95	6.80	10.42	15.37	^*^	20.87	9.0–25.0
34	1	F14	5	19.92	7.79	11.50	18.05	^*^^*^	64.18	4.0–27.0
34	2	F14	2	9.50	6.99	9.82	14.04		31.64	1.0–14.0
34	3	F14	2	8.08	7.41	11.10	16.29		26.69	1.0–21.0

QTL, quantitative trait loci.

In this study, loci with a *P*-value < 0.05 at the experiment-wide level (^*^^*^) are significant at the experiment-wide level. At the chromosome-wide level, loci with a *P*-value < 0.01 (^*^) are defined as significant QTL at the chromosome-wide level, while loci with a *P*-value < 0.05 are suggestive of a QTL. Threshold values were determined via a 10 000 permutation test (Churchill and Doerge 1994). Percent PVE is percentage of phenotypic variance explained, calculated as PVE = 4[1–(MSE_full_/MSE_reduced_)] where MSE_full_ and MSE_reduced_ are the mean squared error of the full model and mean squared error of the reduced model (parameters fixed), respectively (Knott et al. 1996). The 95% confidence intervals were determined with 10 000 bootstraps with replacement.

### Body size QTL

A significant difference in both body weight and length was detected between the offspring from the two half-sib families (*P* < 5.94*10^−18^ for body weight, *P* < 2.83*10^−5^ for length). However, as these families were reared in separate tanks, it is impossible to determine whether this is a genetic effect or simply a confounding environmental effect. To account for this, the residuals from the anova analysis were used for male-effect QTL detection.

Neither male-nor female-linked alleles showed any significant QTL for length at the experiment-wide level. At the chromosome-wide level, one QTL was found to be significant for length in the male-linked alleles on linkage group 6 at 64 cM. An additional four suggestive QTL were found on linkage groups 5, 7, 9, and 14, at positions 69, 95, 86, and 20 cM (Table 4). The four female-linked QTL for length were only suggestive and located on linkage groups 9, 14, 23, and 34 at 5, 44, 60, and 2 cM. These four QTL were all found in F14 (Table 4). These QTL accounted for between 14 and 20% of PVE in the male, and 27 and 34 PVE for female F14.

A total of eight significant and suggestive QTL were found for body weight. Two male-linked QTL were significant at the experiment-wide level (Table 4, Fig. 2). These QTL were located on linkage group 6 at 29 cM and linkage group 9 at 5 cM. The male had one additional QTL significant at the chromosome-wide level, located on linkage group 14 at 20 cM. These QTL accounted for between 19 and 29 PVE (Table 4). Alleles from both females contained QTL for body weight. In F14, there were three suggestive QTL on linkage groups 6, 9, and 34 at 14, 5, and 2 cM. In F13, there were also two suggestive QTL on linkage groups 14 and 18 at positions 74 and 7 cM. These accounted for between 26 and 33 PVE in F14, and 31 and 32 PVE in F13 (Table 4).

## Discussion

Our objective was to use haploids and GBS to rapidly create a dense meiotic map for Chinook salmon to enable QTL analyses and anchor later genomic analyses. We successfully mapped over 3500 RAD-and EST-based SNP loci in a single haploid family, and we then detected thermal tolerance QTL that were isolated to LG 11, LG 16, and LG 34 in corresponding diploid families. Additionally, as size data were readily available, we were able to isolate growth-associated QTL to other linkage groups. Clearly the now mature GBS platforms, in this case coupled with the use of haploids, enable rapid development of genomic resources in organisms for which few exist, providing excellent opportunities to gain new insights into adaptation and genomic architecture (Miller et al. 2012). An improved understanding of the genetic basis of temperature tolerance is especially important for the management and conservation of aquatic species given the expectations of ecosystem warming.

We used paired-end sequencing in multiple individuals to assemble longer contigs in order to annotate our map. Of 3472 RAD markers, 426 (∼12%) were successfully annotated using the SwissProt database (Table S3). This rate of annotation is similar to that in sockeye salmon using similar techniques (∼22%; Everett et al. 2012). Our annotations contained almost 50% transposable elements. This is consistent with other studies in salmonids; transposable elements may have an important role in adaptation (Danzmann et al. 2008). Five markers in the region of our significant QTL had apparently nonremarkable annotations: Three were transposons, one a nuclear encoded ribosomal gene, and one was an EST-based SNP, Ots-104063-132 [GenBank HR308694, NADH dehydrogenase 1 beta subcomplex subunit 2 (Clemento et al. 2011)].

The use of the restriction enzyme *SbfI*, commonly used for RAD sequencing in salmonids, was also intended to increase the rate of annotation (Baird et al. 2008). RAD sequencing generates templates randomly spread throughout the genome, of which only ∼1% might be expected to lie in coding sequences. The enzyme *SbfI* (CCTGCAGG) is 75% GC and has been shown to cut more frequently in coding sequences (Baird et al. 2008; Amores et al. 2011). In zebrafish, 11% of *SbfI* recognition sites are in protein-coding genes, and in stickleback, the figure is 16% (Amores et al. 2011). We do not understand why our annotation rate for protein-coding genes was somewhat lower, ∼6% excluding transposons, although this rate is slightly higher than a similar study by Sánchez et al. (2009) who observed only ∼1.5% in rainbow trout using reduced representation libraries constructed with the enzyme *HaeIII*.

Typically QTL studies of this type require multiple generations with the use of inbred or hybrid lines. Those experimental designs are incompatible with nonmodel species such as Chinook salmon whose anadromous life history makes multigenerational studies extremely difficult. In this study, our use of paired haploid and diploid families to identify and map thousands of markers allowed us to identify QTL within one generation.

Thermal tolerance QTL have been identified in multigenerational study of domesticated salmonids using backcross families derived from captive brood stocks. For example, Jackson et al. (1998) identified two QTL associated with upper temperature tolerance in backcross families of rainbow trout that accounted for between nine and 13% of the phenotypic variance. Two significant QTL for upper temperature tolerance in Arctic charr (*Salvelinus alpinus*) were found to be on homeologs and in a location similar to the QTL markers in the corresponding rainbow trout linkage groups (Somorjai et al. 2003). Comparative studies such as (Somorjai et al. 2003) provide insight into the fate of QTL after the salmonid gene duplication.

The QTL located on our LG 11, LG 16, and LG 34, which correspond to rainbow trout chromosomes 19, 20, and 21 described in Miller et al. (2012) (Tables 3 and:4 Fig. 2), were responsible for up to 64% the observed variance in thermotolerance. The markers associated with the upper temperature tolerance in rainbow trout were found on rainbow trout chromosomes 9, 19, and 25 (Jackson et al. 1998; Palti et al. 2012). While two of our QTL do not appear to overlap those of rainbow trout from the Jackson et al. (1998) study, we did find one corresponding relationship on chromosome 19. A possible explanation for the lack of additional matches could be the differences between our maps and studies. Jackson et al. (1998) used an early map for rainbow trout, composed of fewer markers, so our power to detect QTL will be different. Our experimental approaches for determining temperature tolerance were also different, and the individuals included in our study are not fixed for the traits in question. As a result, QTL in individual families may not be segregating and would not be detected. Finally, different genes may be associated with temperature tolerance in the two species, or the same genes may be on different chromosomes. Recent research in Atlantic salmon suggests that there may be large genetic variation in thermal tolerance even within a species (Anttila et al. 2013).

We observed large variability in the PVE for each QTL among the male and female parents (PVE ranging from 12–64%). While other studies have a fair amount of variability [13–36% (Gutierrez et al. 2012)], none were quite this high. This may be an artifact of our method. Our sample size was relatively low, 100 individuals per half-sib family, and used outbred individuals. Gutierrez et al. (2012) used 120 individuals per family and included five families. Jackson et al. (1998) used between 104 and 144 individuals per family, and the families were based on a backcross breeding design. In our study, the highest PVE values were all in female F14, whose haploid offspring were used to construct the linkage map. The offspring of F14 had the highest percentage of segregating markers as a result. This may also explain the lack of QTL discovered in F13; more markers were segregating for F14, giving more opportunities to discover QTL. Future studies should incorporate multiple haploid families for mapping to diversify the number of segregating markers among families.

### Broader implications

Why is understanding of the genetic basis of adaptation increasingly important? Some species and populations may be able to adjust to climate warming if the change is slow, accumulating over centuries or possibly even several decades. However, many wild populations will less likely adapt in the face of rapid change. Climate records show that the Pacific Northwest corridor of North America is warming, substantially faster than the global average; warming rates of 0.1–0.6°C per decade are anticipated during the coming century (ISAB 2007). These changes will alter snow pack, seasonal stream flows and temperatures, and water quality: all factors that affect locally adapted species including populations of Pacific salmon. Projections suggest that warming will cause major segments of territory for Pacific salmon in North America to become fragmented or inhabitable within just a few decades (Battin et al. 2007; IPCC 2008).

Genetic differences between populations mediate diverse responses to climate change (Peterman et al. 1998; Crozier and Zabel 2006; Rogers and Schindler 2011). Matala et al. (2011) demonstrated that a number of SNPs in Chinook salmon were significantly associated with temperature and that the temperature-associated loci performed better at assigning populations to region and climate than did neutral markers. In Washington State, USA, a population of native Chinook salmon was discovered in Lower Crab Creek, a creek thought unsuitable for their survival. Temperatures in the creek regularly exceed those predicted to be lethal for Chinook salmon, but the habitat was found to support a native, breeding population that is uniquely adapted to survival under the harsh conditions (Small et al. 2011). However, the genetic mechanisms underlying this potential for adaptation remain poorly understood.

We believe that genomic data, especially data that identify adaptively important loci, will provide key information to policy makers who will establish ranking criteria for prioritizing populations for conservation (Clarkson et al. 2012; Hutchings et al. 2012). GBS approaches will provide an efficient mechanism for enabling such data (reviewed in Allendorf et al. 2010). Contemplating such data sets in the context of high-density maps is already providing important insights into the genetics and genomic architecture of adaptively important traits (Hecht et al. 2012; Gagnaire et al. 2013a). However, classical mapping exercises may take two or more generations; the doubled haploid lines used in Miller et al. (2012) were developed over two decades (Parsons and Thorgaard 1984; Lucas et al. 2004). The mapping of haploids as done here provides a comparatively rapid approach to acquire robust genomics data.

Our map already provided a basis to locate genomic regions that are candidates for selection in five population of Chinook salmon from Alaska, although QTL reported here were not implicated (Larson et al. 2013). That study also utilized the linkage relationships of the RAD tags to calculate effective population size, a novel method for nonmodel organisms. It is also important to note that employing haploids for mapping enables the immediate categorization of paralogous sequence variants in the duplicated salmonid genome (Spruell et al. 1999), a factor that greatly complicates interpretation of next-generation genotyping data (Seeb et al. 2011).

We believe that the use of GBS coupled with haploid mapping will expedite applications of genomic information by conservation and management agencies. Many interested in salmonids are already aggressively seeking genomics data to improve understanding of adaptively important loci (Hale et al. 2013; Hecht et al. 2013; Larson et al. 2013).

## Data archiving statement

Data for this study are available at the NCBI BioProject database under Accession number PRJNA232256.

## Supporting Information

**Table S1.** EST Assays Included in the linkage map and used to screen haploid individuals. **Table S2.** Sequencing libraries. **Table S3.** Linkage map in table format. **Table S4.** Annotations for RAD tags included in the linkage map. **Table S5.** Additional QTL data.
